# The Potential for Microalgae as Bioreactors to Produce Pharmaceuticals

**DOI:** 10.3390/ijms17060962

**Published:** 2016-06-17

**Authors:** Na Yan, Chengming Fan, Yuhong Chen, Zanmin Hu

**Affiliations:** 1 Institute of Genetics and Developmental Biology, Chinese Academy of Sciences, Beijing 100101, China; yanna19881204@163.com (N.Y.); cmfan@genetics.ac.cn (C.F.); yhchen@genetics.ac.cn (Y.C.); 2 University of Chinese Academy of Sciences, Beijing 100049, China

**Keywords:** microalgae, bioreactor, pharmaceuticals, recombinant proteins

## Abstract

As photosynthetic organisms, microalgae can efficiently convert solar energy into biomass. Microalgae are currently used as an important source of valuable natural biologically active molecules, such as carotenoids, chlorophyll, long-chain polyunsaturated fatty acids, phycobiliproteins, carotenoids and enzymes. Significant advances have been achieved in microalgae biotechnology over the last decade, and the use of microalgae as bioreactors for expressing recombinant proteins is receiving increased interest. Compared with the bioreactor systems that are currently in use, microalgae may be an attractive alternative for the production of pharmaceuticals, recombinant proteins and other valuable products. Products synthesized via the genetic engineering of microalgae include vaccines, antibodies, enzymes, blood-clotting factors, immune regulators, growth factors, hormones, and other valuable products, such as the anticancer agent Taxol. In this paper, we briefly compare the currently used bioreactor systems, summarize the progress in genetic engineering of microalgae, and discuss the potential for microalgae as bioreactors to produce pharmaceuticals.

## 1. Introduction

The demand for recombinant proteins is growing globally because of their great application values in industry, diagnosis and therapy. The bioreactor systems that are used to produce recombinant proteins are becoming more important for producing large quantities of proteins, particularly those that have associated limitations due to cost or traditional source availability. Based on different types of organisms, several novel bioreactor systems were recently developed due to the advance in biotechniques. In general, a particular bioreactor system may be selected for a specific protein production based on costs as well as the integrity, purity and expression level of the protein.

Currently, recombinant proteins can be expressed in bacteria, yeasts, mammalian cell lines, transgenic animals and plants, and each of these bioreactor systems has specific advantages and limitations [1,2,3]. As a source of natural products, bacteria and yeasts have long been employed in the production of small and simple recombination proteins at a relatively low cost [1,4]. However, bacteria have no post-transcriptional and post-translational modifications, such as intron splicing, multimeric protein assembly, glycosylation and disulfide bond formation, and processes that are essential for generating functional eukaryotic proteins [5,6]. Furthermore, heterologous proteins, expressed in intracellular with high levels, readily aggregate and form insoluble inclusion bodies that require expensive downstream processing [1,2,3]. Despite the ability of yeasts to carry out post-translational modifications, the protein glycosylation pattern usually involves hyperglycosylation, which does not reflect what occurs in higher organisms [7].

Mammalian cell lines are frequently used to produce proteins for therapeutic use; indeed, the USA has approved many different therapeutic and diagnostic proteins that are produced in mammalian cell lines [8,9]. However, oxygen deficiency, the accumulation of waste product or sensitivity to stirring forces can result in instability of mammalian cells, making it difficult to culture these cells in large volumes [9,10,11]. In addition, high levels of complex proteins can be obtained from the milk of transgenic animals [12]. Regardless, the greatest disadvantage of mammalian bioreactor systems is contamination of agents and oncogenic sequences, which can cause diseases [13].

Compared with the above-mentioned expression systems, transgenic plants provide numerous advantages, including the capacity to produce functional recombinant proteins at high levels in an inexpensive and safe manner. Furthermore, the scaling-up process for plants can more easily meet demands by utilizing existing agricultural processes (“molecular farming”), and the input is lower in contrast with other systems [14]. As eukaryotes, plants are able to perform post-translational modifications; similar to animal cells, plant cells have pathways for protein synthesis and folding, and protein secretion and modification, which are required for pharmaceutical protein bioactivity [15]. More importantly, plants cannot be infected by the major human pathogens, which may contaminate the systems of bacterial and mammalian [16]. The expression levels of total soluble proteins (TSPs) obtained from transgenic plants range from 0.001% to 46.1% [17,18], and the most of plants-produced human therapeutic proteins display structural, biochemical and functional properties that are very similar to proteins in humans and animal cell culture-produced proteins [19,20].

Plants produce many types of proteins, including enzymes [21], hormones [22], functional vaccines [17,23], antibodies [24,25], and a variety of other therapeutic proteins [26]. Examples of pharmaceutical proteins that are being expressed in plants include human growth hormone, human serum albumin, interferons, and erythropoietin [27]. Several recent reviews describe the significant advances in this field [28,29,30,31,32]. The first Food and Drug Administration (FDA)-approved plant-made biopharmaceutical (PMB) is glucocerebrosidase; this injectable product produced in carrot cells is used to treat a genetic/metabolic disorder [33]. Another major advancement is the application of chloroplast-generated biopharmaceuticals for toxic antibody suppression [29]. Chan and Daniell noted the challenges of plant cells-produced vaccine antigens toward clinical development, though these oral vaccines may be quite attractive for both human and veterinary purposes [31,34].

Nonetheless, several disadvantages have hindered the use of plants as successful recombinant protein-producing system. Firstly, plants have different types of glycosylation patterns compared with animal cells, which may alter the function of the recombinant protein or even decrease immunogenicity; The second problem includes the controversial issues of regulations and safety, especially with regard to the risk of gene flow via transgenic pollen. Studies on gene flow between transgenic plants and native races have been reported, such as genes that encode *Bacillus thuringiensis* proteins in corn [35] and herbicide resistance genes in canola [36]; Third, much time is required from the transformation step to the acquisition of a purified protein. Moreover, the purification of proteins from plants is inconvenient because they cannot be secreted [37].

Microalgae are a diverse photosynthetic group, consisting of eukaryotic organisms and prokaryotic cyanobacteria. Microalgae have unique advantages, including a high growth rate, ease of cultivation, low growth costs and metabolic pathways that are similar to those of higher plants, leading to the same post-transcriptional and post-translational modifications that occur in higher plants [38,39]. Indeed, as photoautotrophic sunlight-driven cell factories, microalgae provide an efficient means of converting solar energy into biomass, producing fatty acids, lipids, vitamins, carbohydrates, antibiotics, antioxidants and proteins [40,41].

Moreover, microalgae are valuable sources of novel biologically active products, and because their growth requires only inexpensive substrates, these cells can serve as economical and effective bioreactors for obtaining high added-value compounds for healthy food [42,43,44]. Microalgae, such as *Spirulina maxima*, *Synechococcus* sp., *Scenedesmus obliquus*, *Porphyridium cruentum*, *Dunaliella salina*, *Chlorella vulgaris*, *Chlamydomonas reinhardtii*, and *Anabaena cylindrica* contain valuable nutrient compounds. However, microalgal cultivation began only several decades ago [45,46].

In addition to naturally produced compounds, microalgae are also a promising platform for producing recombinant proteins and other valuable natural products, such as cosmetics, pharmaceuticals, biofuels and health supplements [47,48,49,50], providing an attractive alternative to the current bioreactor systems. Compounds that are synthesized by microalgae include growth and blood-clotting factors, immune regulators, hormones, enzymes, monoclonal antibodies, viral vaccines, and other natural products, such as the anticancer agent Taxol. Table 1 roughly summarizes the advantages and disadvantages of bacteria, yeasts, cultured mammalian cells, animals, plants and microalgae for production purposes. This paper reviews the status of microalgae as bioreactors that produce pharmaceuticals.

## 2. Brief Introduction to Microalgae

Algae include the microscopic (microalgae) and macroscopic forms (macroalgae) forms, with the latter largely consisting of seaweed. It has been estimated that there are one to ten million algae species, most of which are microalgae [53]. Culturable microalgae can be classified into four categories: cyanobacteria, green algae, chrysophyte and red algae. Cyanobacteria, also known as blue-green algae, are distinguished from other bacteria because they perform oxygenic photosynthesis.

### 2.1. Growth Characteristics

An obvious and tangible diversity can be observed among microalgae. Their growth modes include phototrophy, photoheterotrophy, and heterotrophy, and each growth mode can be either obligate or facultative [54].

Compared with plants traditionally used for production, microalgae have several advantages with respect to their growth characteristics. Microalgae do not require fertile soil; because microalgae can use nutrients quite efficiently, a medium that only contains essential nutrients can be used, thus avoiding water pollution due to unused fertilizers. Furthermore, some microalgae can grow in brackish water, saline water or seawater, which can help to conserve limited freshwater resources.

Microalgae photosynthesize under conditions of high light and low temperature. However, lack of essential nutrients, such as nitrogen, can result in starvation conditions, and nitrogen starvation leads to the cessation or slowing down of cell division, which then results in reduced utilization of photosynthetic products [55,56,57]. Based on this, nitrogen starvation had been employed to manipulate the mass-cultivated microalgal metabolism to obtain lipids or carbohydrates-rich biomass [58].

### 2.2. Nutrient Value

Microalgae can improve the nutritive value of conventional food and animal feed and can effectively improve the health of humans and animals [59]. As sources of protein, various microalgae are unconventional and contain a high protein content [60]. The patterns of amino acids have advantages over that of other food proteins [61]. Carbohydrates in microalgae exist in the forms of sugars, starch, glucose, and other polysaccharides, which are very easy to digest. Thus, whole dried microalgae can be used as supplements in food or feed with no limitations [62].

The lipid content in some microalgae can account for 90% of the dry weight under certain conditions, with an average lipid content of 1% to 70% [63]. As an essential component of lipids, fatty acids of the ω3 and ω6 families in microalgae are of special interest. Microalgae contain nearly all essential vitamins, such as nicotinate, biotin, folic acid and pantothenic acid [64,65,66,67]. Moreover, there are large amounts of pigments, such as chloroiphyll, phycobiliproteins and carotenoids, in microalgae cells.

The production of nutritional supplements from microalgae has been a major research area in microalgal biotechnology for many years. Products generated from dried biomass or cell extracts of *Chlorella*, *Dunaliella* and *Spirulina* are marketed as nutraceuticals or health food and are sold for many hundreds of thousands of dollars [68,69].

### 2.3. Genetic Research on Microalgae

Genetic research on microalgae has achieved great progress to date. The Calvin cycle was discovered through *Chlorella* research in 1948 [70]. The genome sequences ofseveral microalgae including *Cyanobacteria* [71,72], *Cyanidioschyzon merolae* [73], *Thalassiosira pseudonana* [74], and *C. reinhardtii* [75] are publicly available. In addition, the genome sequence and genetic transformation of *Chlorella pyrenoidosa* [76] and the oleaginous alga *Nannochloropsis gaditana* [77], were reported.

*C. reinhardtii* is a model organism in microalgal genetics research, and much progress has been achieved using this organism in recent years. Fu *et al.* found that *N*^6^-methyldeoxyadenosine, a DNA modification, is an active transcriptional start sites in the genome of *Chlamydomonas* [78]. Li *et al.* established an indexed, mapped *C. reinhardtii* mutant library that enables reverse genetic studies of biological processes in this species [79]. Yang *et al.* described the disruption of *C. reinhardtii* ferredoxin-5 (FDX5) can lead to a dark growth deficiency and influence the membrane ultrastructure and membrane lipids [80], and Dejtisakdi and Miller found that overexpression of *C. reinhardtii* fructose 1,6-bisphosphatase (CrFBPase) has a negative effect on growth [81]. A mutation of starch deficiency in *Dunaliella tertiolecta* resulted in increased triacylglycerol content in the cells [82], and the genomic basis for the Starch-to-Lipid Switch has been investigated in *Chlorella* [76]. A new nuclear transformation method by microparticle bombardment was developed in *Phaeodactylum tricornutum* using PCR-amplified DNA fragments [83]. In addition, Ferreira-Camargo *et al.* found that selenocystamine can improve accumulation of disulfide bonds-contained proteins in green algal chloroplasts [84].

These studies helped to lay the foundation for research on microalgal applications, including genetic engineering.

## 3. Genetic Engineering and Production of Pharmaceuticals and Recombinant Proteins

### 3.1. Microalgal Transformation

As a platform for producing recombinant protein, the effective utilization of transgenic microalgae relies on stable transformation systems, including vector construction and transformation methods. Recombinant proteins are typically expressed by the genome of nucleus or chloroplast, which have important differences (see Table 2). In the past 20 years, many species of microalgae were successfully transformed, most of which have been achieved via nuclear transformation [85].

Typical methods for microalgal transformation are based on transient permeabilization of the cell membrane, which enables plasmid DNA to pass through the membrane and enter the cell. There are many methods for cell permeabilization. One method is to vortex the cells with the DNA, polyethylene glycol (PEG) and glass beads [86]; this is simple, efficient, and suitable for transforming wild-type *Chlamydomonas* after enzyme-mediated cell wall degradation. Another method of *Chlamydomonas* transformation employs silicon carbide (SiC) whiskers, an approach that does not require removal of the cell wall [87] and has been used to manipulate several microalgae, such as *Symbiodinium* and *Amphidinium* [88]. However, SiC whiskers are harmful to human health and should not be the first choice. Electroporation was introduced for transferring genes into many types of cells and organisms, such as thin-walled cells, cell wall-reduced mutants, protoplasts, and naked cells, and is used to obtain stable *Chlamydomonas* transformants with high efficiency [89,90]. In this method, the lipid bilayer is temporarily disrupted by an electronic pulse and DNA molecules are allowed to pass into the cell. Using this technique, the transformation of *C. reinhardtii*, *D. salina*, *C. vulgaris*, *Ostreococcus tauri*, and the red algae *Cyanidioschyzon merolae* has been successfully achieved. Microparticle bombardment is the preferred method of transformation for previously untransformed species of microalgae. Viruses, which infect brown algae and Chlorella-like algae, can also be used in microalgal transformation [91,92], but this approach requires more investigation. In addition, the use of artificial transposons has expanded to the application of genetic transformation. Artificial mini-transposons contain elements of natural transposons that are essential for transposition, and a mini-transposon-transposase complex has been introduced into *Cyanobacterium spirulina* [93]. Although eukaryotic microalgal transformation has not been reported to date using this method, the transposon approach may also be suitable for eukaryotic microalgae when no host factor is required. Particle bombardment is the most frequently used method for transformation of plant tissues and cells as well as prokaryotes; regarding microalgae, it has been successfully used in diatoms [94,95,96,97]. Organelles can also been transformed using this method, such as chloroplasts and mitochondria [98,99,100,101].

Chloroplast transformation has also been reported in the unicellular red algae *Porphyridium* sp. [98]. The chloroplast transformation system of *Chlamydommonas* is the first and best studied in green algae [86,102,103]. *Chlamydommonas* is an excellent model system for research because its genome has been sequenced [104]. Many microalgal species have been stably transformed to date. A luciferase (Luc) and a homologous nitrate reductase gene have been transformed into *Chlorella* [105]. Five heterololgous genes were transformed into the chloroplast of *D. tertiolecta*, and the recombinant enzymes were produced in a stable manner [106]. Other economically valued chlorophytes, such as *Haematococcus pluvialis*, have been stably transformed through the development of a novel chloroplast expression vector [107]. A food source in aquaculture, diatoms with silica-based cell walls, can also be used to produce therapeutic proteins, and six species, *Phaeodactylum tricornutum* [94], *Cyclotella cryptica* [95], *Navicula saprophila* (pennate diatoms) [95], *Cylindrotheca fusiformis* [108], *Thalassiosira weissflogii* [96] and *Thalassiosira pseudonana* [109], as well as *Chaetoceros* sp. (centric diatoms) [110], have been transformed using the microparticle bombardment. Dinoflagellates, an important phytoplankton, are unicellular eukaryotic alveolar algae and some species have typical prokaryote characteristics and eukaryotic features. Among this group, *Amphidinium* and *Symbiodinium* have been successfully transformed [88].

The application of selectable markers is essential for efficient transformation of microalgae. Several dominant and recessive markers have been investigated. Recessive markers have been chosen by functional complementation of the corresponding endogenous genes and mutants [111]. Among these, nitrate reductase has been employed in *C. reinhardtii* [112], *Volvox carteri* [113], *Chlorella sorokiniana* [105], and *Dunaliella viridis* [114]. Dominant markers are the most widely used selectable markers that confer resistance to antibiotics or herbicides. In *Chlamydomonas*, *aadA* is used for resistance to spectinomycin and streptomycin [115], *aphA*6 for resistance to kanamycin, *aph*7 for resistance to hygromycin B, the *aphVIII* for resistance to paromomycin [116], *ble* for resistance to zeomycin and phleomycin [117], the 16S ribosomal gene for resistance to spectinomycin; a mutated *als* gene also confers resistance to sulfonylurea herbicides [118]. All of these markers provide effective, stable antibiotic resistance. In addition to *Chlamydomonas*, the *ble* gene can also be used in *Volvox carteri* [119], *Phaeodactylum tricornutum* [94,96], and *Cylindrotheca fusiformis* [108]. Additionally, the *nptII* gene can be used in *Navicula saprophila*, *Cyclotella cryptica* [95], in the dinoflagellates *Amphidinium* sp. and *Symbiodinium microadriaticum* [88], in *Chlorella* [76,120,121] and in the diatoms *Phaeodactylum tricornutum* [97]. Different microalgal species exhibit diverse antibiotic sensitivities due to the presence of different marker genes [96,97].

The promoter is an important aspect of successful microalgal transformation. For nuclear transformation in *P. tricornutum*, likely promoter choices are those of *fcpB* encoding chlorophyll a/c binding protein B of fucoxanthin [94,97,122], and nitrate reductase, which is activated when nitrate is the nitrogen source in the medium [123,124,125,126,127]. The CaMV35S promoter is also efficient in microalgae, such as *C. ellipsoidea* [128,129,130], *C. vulgaris* [131], *Haematococcus pluvialis* [132], *D. salina* [133,134,135] and *C. reinhardtii*. However, the expression level in *C. reinhardtii* is low. The RbcS2 promoter of *C. reinhardtii* has successfully been used in *C. ellipsoidea* for transient expression of the resistance gene or recombinant protein [128,131]. The maize *Ubiquitin* (*Ubi*1) gene promoter can be used for stable expression of NP-1, an α-defensin encoded by a rabbit gene, in *C. ellipsoidea* [120]. The tobacco mosaic virus (TMV) promoter enhances translation efficiency [130], and in *D. salina* the ubiquitin-Ω promoter successfully drives expression of hepatitis B surface antigen [133]. With regard to chloroplast transgenes, the *psbA*, *psbD* and *rbcL* promoters are typically used in *C. reinhardtii*. Recently, Seo *et al.* indicated that the EF2 promoter of *P. tricornutum* can be utilized for the expression of target gene in *P. tricornutum* and *C. reinhardtii* [136].

Many species of microalgae had been successfully transformed; however, stable *Chlorella* transformants are lacking for a long time. *Chlorella* is an important genus in which a few species of microalgae can be used for industrial production, and genetic transformation of this group has important significance for employing *Chlorella* as a biological reactor. Although foreign gene transient expression has been successfully achieved in *Chlorella*, *C. sorokiniana* and *C. vulgaris*, none was able to produce target product [129,131,137]. Flounder growth hormone (fGH) was produced in protoplasts of *C. ellipsoidea*, and it was accumulated to 400 mg/L estimated by ELISA [128]. In our previous work, NP-1 was successfully expressed in *Chlorella* [130], but the lines stably expressing NP-1 were not obtained because when the selection antibiotic G418 was removed, the NP-1-expressing cells were lost [138]. Nonetheless, stable expression of foreign genes in transgenic *Chlorella* was achieved with technique improvement. For example, as stated above, a luciferase (Luc) gene and homologous nitrate reductase gene have been stably transformed into *Chlorella* [105,129,139]. Defensin NP-1 can be produced in *C. ellipsoidea* [120], and expression of GmDof4 in *C. ellipsoidea* can significantly increase the total lipid content. In *Chlorella pyrenoidosa*, overexpression of an *Arabidopsis thaliana* nicotinamide adenine dinucleotide (NAD(H)) kinase increased the lipid content of cells by 110.4% without effects on the growth rate [76]. Recently, a new inducible expression system has also been developed for *Chlorella vulgaris* [140].

Nuclear transgenic expression in microalgae is occasionally inefficient, possibly due to gene silencing [141,142,143]. Furthermore, exogenous genes might not be expressed properly if the transgenic clones are not maintained under constant selection [144]. With regard to the general feasibility of expressing foreign proteins in microalgae, it appears that despite the considerable body of knowledge accumulated, inducing high expression of transgenes is difficult, possibly due to epigenetic mechanisms [144]. Other reasons for transient expression may include the following: (1) the heterologous genes might be not actually inserted into the genome, which can be detected by Southern hybridization, and Southern hybridization results were not provided in most of the papers reporting the transient expression of foreign genes in microalgae [129,145,146]; (2) the selected transgenic colony contained non-transgenic cells, and without constant selection, the number of transgenic cells may have decreased or these cells may have disappeared. In our work on NP-1 expression in *C. ellipsoidea*, at least five rounds of continuous selection were performed to obtain stable transgenic lines [120]. Therefore, to obtain stable transgenic *Chlorella*, a long period of continuous selection of individual clone is necessary.

### 3.2. Genetically Engineered Microalgae

Microalgal biotechnology has achieved important advances in recent years due to the development of new technologies [147], and several examples in biotechnological applications of engineered microalgae have shown significant promise. In diatoms, Genetic engineering is an important approach of microalgae to produce particular lipids and bio-diesel [148]. In *C. ellipsoidea*, overexpression of GmDof4, a soybean transcription factor, significantly increased the lipid content by 46.4% to 52.9% [121]. In addition, researchers found that the expression of a foreign metallothionein gene can enhance the binding capacity to metals and the moth bean pyrroline-5-carboxylate synthetase (P5CS) gene expression can result in the accumulation of free proline in microalgae [149,150].

Most of the advances in genetic engineering of microalgae have been realized in *C. reinhardtii* through the use of both nuclear and chloroplast transformation. *C. reinhardtii* is a good bioreactor for the production of many important recombinant proteins, including antibodies, protein therapeutics, vaccines, enzymes and additives, such as anti-CD22-gelonin single chain, viral protein 28 (VP28), erythropoietin, phytase (see Table 3). A summary of information on the production of recombination proteins in *C. reinhardtii* and several other important microalgae is provided in Table 3.

In addition to *C. reinhardtii*, several other microalgae have been studied and are considered more robust and more productive or are generally recognized and certified as safe, including species of *Chlorella*, *Scenedesmus* and *Dunaliella*. Although information about recombinant protein production in these species is lacking, in some cases, they can produce recombinant proteins at the same levels as *C. reinhardtii*. For example, five chloroplast industrial enzymes were produced in *D. tertiolecta*, accumulating in the same amounts as in *C. reinhardtii* [106], and recombinant protein expression in *Scenedesmus dimorphus* has achieved the same results [179]. Thus, other species of microalgae also have the ability to produce recombinant proteins [179]. For instance, a monoclonal human IgG antibody against hepatitis B has been expressed in the diatom *P. tricornutum* [123,126]. Because the codon usage of *P. tricornutum* is similar to that of humans [180], this species could be beneficial for the production of biopharmaceuticals.

Additionally, successful *Porphytridium* spp., *Euglena gracilis* and *Haematococcus pluvialis* chloroplast transformation has also been reported [98,100,107].

### 3.3. Production of Pharmaceuticals and Therapeutic Proteins

#### 3.3.1. Subunit Vaccines

As a platform for the production of subunit vaccines, transgenic microalgae have attracted considerable attention [181]. Potential vaccine candidates have been produced against viruses and bacteria as well as malaria and other communicable diseases or have been investigated for non-viral diseases.

The E2 protein, which constitutes an antigen for vaccines against classical swine fever virus (CSFV), can be produced in *Chlamydomonas* chloroplast. The E2 protein can accumulate to 1.5%–2% of TSP [163], and crude extracts of E2-transformed algae have been administered via subcutaneous injection and orally immunization in mice; the former resulted in a significant level of serum antibody against CSFV, whereas the later caused no immune response to E2.

Foot-and-mouth disease virus (FMDV) is an important virus that affects livestock. To produce a vaccine against FMDV in *C. reinhardtii*, the VP1 protein was fused with a potent mucosal adjuvant, cholera toxin B subunit (CTB). VP1-CTB can accumulate to 3% of TSP [154] and exhibited binding affinity towards GM1-ganglioside, separately acting as an antigen for FMDV VP1 and CTB proteins, separately.

The gene that encodes the hepatitis B virus surface antigen (HBsAg) was successfully transformed into the green alga *D. salina* by electroporation, and stable nuclear transformants were obtained [133]. Western blot analysis demonstrated HBsAg production, with levels of 1.6–3.1 ng/mg of total protein in different transformants with different insertion sites.

White Spot Syndrome Virus (WSSV) causes severe damage to shrimp farms worldwide. The main viral envelop protein of WSSV, Viral protein 28 (VP28), is a target for subunit vaccines [182]. The gene encoding the VP28 protein was successfully transformed into the nuclear genome of *D. salina* and the chloroplast of *C. reinhardtii* [164,174], with an output of 21% of total protein in the former case [164], and approximately 3 ng/mg of total protein in the latter [174]. Under the challenge of WSSV, the survival rate of Ds-VP28-vaccinated crayfish was 41%, much higher than the control group, which had a mortality rate of 100%. This indicated that oral administration of VP28-transformed algae can protect crayfish against viral pathogens.

*Staphylococcus aureus* is a bacterium that can cause skin infections, respiratory diseases and food poisoning. The fused protein of D2 fibronectin-binding domain of *S. aureus* and the CTB mucosal adjuvant was stably expressed in the chloroplast of *Chlamydomonas* [166]. The fused D2-CTB protein was accumulated to 0.7% of TSP, and oral administration to mice caused both systemic IgG and mucosal IgA responses. Oral vaccination of mice with D2-CTB protected against a lethal dose of *S. aureus*, which is the first demonstration of the efficacy of an oral vaccine produced in algae.

Vaccines have been developed to prevent or treat cancers. The FDA has approved Gardasil and Cervarix as cancer vaccines to protect against human papillomavirus (HPV) types 16 and 18, which is the arch-criminal of about 70% of cervical cancer cases. A transgene encoding the E7 oncoprotein of HPV-16 was transformed to *C. reinhardtii* and accumulated to 0.12% of TSP [155]. The experiment of subcutaneous injections showed that protection with high levels were achieved after the E7 protein expressed in a tumour cell line, which indicated that green microalgae can be used as a viable platform to produce cancer vaccines.

Glutamic acid decarboxylase-65 (GAD65) is a major autoantigen for human type 1 diabetes, which is an autoimmune disease resulting from the destruction of insulin-producing beta cells in the pancreas. By immunizing non-obese diabetic (NOD) mice, studies on type 1 diabetes found that GAD65 can prevent or delay diabetes onset [183]. The expression level of human GAD65 in the chloroplasts of *C. reinhardtii* can reach 0.25% to 0.3% of TSP [165], and the algal-derived hGAD65 reacts with diabetic sera and has the ability to induce the proliferation of spleen lymphocytes from NOD mice.

Many other vaccines have been expressed in microalgae, such as *C. reinhardtii*-produced malaria vaccines [167,169] that fuse the algal starch matrix protein called granule-bound starch synthase (GBSS) with apical major antigen (AMA1) and major surface protein (MSP1), two clinically relevant malaria antigens, separately. Experiments showed a significant reduction in parasitemia and an increased life span, and the immune sera from GBSS-MSP1 immunized mice resulted in strongly protection against malarial infection [167].

One strategy for malaria eradication includes the combination of transmission blocking vaccines (TBVs) for preventing the disease spread with drug-based therapies. As potential TBVs, Pfs48/45, Pfs25, and Pfs28, which are difficult to produce in traditional expression systems, were transformed into *Chlamydomonas* [168,169]. The transmission of *Plasmodium* oocytes can be completely blocked by antibodies against algal-expressed Pfs25. Mice fed freeze-dried Pfs25-CTB produced in algae displayed a mucosal IgA response to Pfs25 and CTB, and elicited serum IgG antibodies against CTB. Because no IgG antibodies of Pfs25 were elicited, malaria transmission couldn’t be blocked. However, this study does support that the transgenic algae are promising to produce oral vaccines against mucosal infections [170,179].

Immunotherapy is a promising alternative to conventional drug therapy for hypertension. A chimeric protein, angiotensin II fused with a nucleocapsid antigen of hepatitis B virus (HBcAg) was produced in *C. reinhardtii*. The expression levels of this fused protein reached 0.05% of TSP in several transgenic lines; but the immunogenic properties of antigen derived from the HBcAgII algae still need to be assessed [176].

By modifying gene codons, HIV antigen P24 was expressed in *Chlamydomonas*, with the level of recombinant protein accounting for up to 0.25% of the total cellular protein [177]. In addition, a new recombinant protein that is a chimaera fused the CTB mucosal adjuvant with the p210 epitope of ApoB100 (CTB:p210) was expressed in the chloroplasts of *C. reinhardtii* as an approach to create an oral vaccine against atherosclerosis. The production of this fusion protein can reach 60 μg/g of fresh algae weight. The immunogenic activity was tested by orally administration in BALB/c mice, and elicited anti-p210 serum antibodies, which can last for at least 80 days [178].

#### 3.3.2. Antibodies, Immunotoxins, Antimicrobial Agents and Others

Monoclonal antibody is an effective therapy for the treatment of various human diseases. The first mammalian protein produced in the chloroplast of microalgae was a monoclonal antibody protecting human against glycoprotein D of herpes simplex virus (HSV). This large single-chain (lsc) antibody, which is a fusion protein of the entire IgA heavy chain and the light chain variable region via a flexible linker, was expressed in *C. reinhardtii* chloroplast [151].

Immunotoxins, consisting of a toxin molecule and an antibody domain, can bind to target cells and inhibit its proliferation, thus can be used to kill cancer cells in anti-cancer therapies. Using a single-chain antibody fragment targeting a B-cell surface epitope CD22, fused with exotoxin A enzymatic domain from *Pseudomonas aeruginosa*, Tran *et al.* found that both the monovalent and divalent immunotoxins can be produced and assembled in chloroplast of microalgae [153]. Furthermore, both immunotoxin molecules can significantly inhibit tumour growth in animal experiments, improving mouse survival rate in the assay of tumour-challenge [153]. Similar results were observed when gelonin, a different eukaryotic toxin from *Gelonium multiflorum*, was fused to monovalent and divalent anti-CD22 single-chain antibodies [152].

Defensins, a kind of small cationic peptides, can be used as promising alternative to antibiotics. For example, mature rabbit neutrophil peptide 1 (NP-1), an α-defensin with a broad anti-microbial activity that can defend against gram-negative or positive bacteria, certain viruses, and pathogenic fungi, has been successfully transformed into *C. ellipsoidea* [120].

Flounder growth hormone (fGH) can be expressed in *C. ellipsoidea* and accumulate to approximately 400 μg per liter. Flounder fry fed transgenic *Chlorella* increased by 25% in length and width within 30 days [128].

Several types of enzymes have been successfully produced in microalgae. A gene from *E. coli* encoding a bioactive phytase enzyme (AppA) was expressed in *C. reinhardtii*, and AppA algae can significantly reduce the phytate content and increase the content of inorganic phosphate in chick manure when used as a direct food additive [171]. Moreover, bioactive xylanase and two α-galactosidases have been expressed in the chloroplasts of *C. reinhardtii* and *D. tertiolecta* [106]. In particular, *C. reinhardtii* nuclear genome transformation resulted in more than 100-fold the xylanase activity when xylanase was fused to a secretion signal, self-processing viral peptide (2A), and the selection marker gene *ble* [172].

We summarized the potential pharmaceuticals and recombinant proteins that were produced from microalgae and tested in animal experiments in Table 4.

## 4. Discussions and Conclusions

Microalgae are important natural resources that contain valuable proteins, oils, fatty acids, polysaccharides and other bioactive proteins that are currently commercially available. Moreover, as a new type of bioreactor, microalgae can produce recombinant pharmaceutical proteins, such as vaccines and antibodies. In this review, we outlined significant potential avenues for microalgal production of pharmaceuticals.

Subunit vaccines are mainly produced and delivered from expensive injection administration of purified protein. In the microalgal production of pharmaceuticals, the use of microalgae to produce oral vaccines could be highly promising. It had been proved that several vaccine antigens can accumulate and correctly fold in microalgae, and some fusion proteins with increased antigenicity can be effectively delivered orally. These give a potential alternative way to produce subunit vaccines by making oral tablet of microalgae inexpensively with systemic and mucosa immune reactivities [181]. A better acknowledgement of the immune responses of adjuvants and orally proteins will promote the development of oral vaccines significantly [184].

According to advances in microalgal research, it appears that *C. reinhardtii* is the most promising microalgae for producing pharmaceuticals. In addition to *C. reinhardtii*, other microalgae have been preliminarily considered as bioreactors for pharmaceutical production, but some species, such as *Chlorella spp*. and *Phaeodactylum tricornutum*, may be more promising due to recent significant achievements in research of their genomes [83,146,185,186]. Indeed, these microalgae have advantages compared with *C. reinhardtii*, such as ease of cultivation, edibility (*Chlorella*), rapid growth, and ease of industrialized production. Nevertheless, *Chlorella* is small in size and possesses a resilient cell wall; thus, utilization of *Chlorella* for recombinant protein expression is hampered by a lack of genetic tools. Although several reports have shown stable expression of transgenes in *Chlorella* [76,120,129,139], achieving stable transformation in *Chlorella* is still difficult compared to many other microalgae due to unstable nuclear integration, position effects, inefficient selection of transgenic cells, inefficient transcription of heterologous genes, inaccurate RNA processing, and the bias of codon usage. In addition, transformed strains are easily lost during passaging once the selective pressure has been removed [187]. Therefore, the development of sophisticated genetic tools such as a powerful expression box would be helpful for nuclear and chloroplast transformations in *Chlorella* genetic engineering.

With regard to plants, after decades of work, the first biopharmaceutical-taliglucerase alfa, which was produced in cultured carrot cells, was approved by the FDA of the United States for treatment with Gaucher’s Disease in 2012 [188,189,190,191,192]. Several plant-based oral vaccines have been assessed in clinical trials to determine their safety and immunogenicity [193,194,195,196]. Despite the many advantages for pharmaceutical production and the significant advances, especially for *C. reinhardtii*, the microalgal approach for bioreactors is still in its infancy. To date, no pharmaceuticals produced from microalgae have obtained approval for commercial production or are under clinical trial, and only a few subunit vaccines/recombinant proteins were tested in animal experiments (see Table 4). Therefore, extensive work remains to be performed before microalgae can be used for large-scale production of pharmaceuticals for commercial use.

The following aspects should be given more attention in future work: (1) transgenic microalgal safety evaluations according to the regulations of different countries; (2) production standards for recombinant pharmaceutical proteins under GMP conditions, including transgenic algal culture at a large scale, algal cell processing, protein purification, structural and function characterizations and product quality control; (3) preclinical trials to verify product safety and efficiency; and (4) clinical trials.

“The application of algal systems to edible vaccines is increasingly gaining interest”, comments from Christoph Griesbeck. “Aside from biopharmaceuticals, an additional application of algae for pharmaceutical products could involve novel metabolites improved by metabolic engineering” [197]. With the development of new techniques for molecular manipulation and metabolic engineering, advances in microalgal approaches will inevitably be achieved, and the significant potential of these organisms as routine expression systems for the manufacture of pharmaceuticals will be realized in the near future.

## Figures and Tables

**Table 1:** Rough comparison among different expression platforms to produce pharmaceuticals (modified from [51,52]).

Expression Systems	Bacteria	Yeasts	Cultured Mammalian Cells	Animals	Plants	Microalgae
Protein folding accuracy	Low	Medium	High	High	High	High
Glycosylation	None	Incorrect	Correct	Correct	Minor Differences	Minor Differences
Product quality	Low	Medium	High	High	High	High
Protein yield	Medium	High	High	High	High	High
Production scale	Limited	Limited	Limited	Limited	Worldwide	High
Production time	Short	Medium	Long	Long	Long	Short
Scale-up cost	High	High	High	High	Medium	Low
Overall cost	Medium	Medium	High	High	Low	Low
Contamination risk	Endotoxins	Low	High	High	Low	Low
Safety	Low	Unknown	High	High	High	High
Storage cost	Moderate	Moderate	Expensive	Expensive	Inexpensive	Low
Distribution	Medium	Medium	Difficult	Difficult	Easy	Very easy
Reproduction	Easy	Easy	Difficult	Medium	Easy	Very easy

**Table 2:** Differences between the genome engineering of the nucleus and chloroplast.

Genome Engineering	Nucleus	Chloroplast
Gene expression machinery	Eukaryotic	Prokaryotic
Protein localization	Cytoplasm, nucleus, chloroplast, ER, mitochondria, secretion	Chloroplast
Modifications	Phosphorylation, glycosylation, disulfide bond	Phosphorylation, disulfide bond
Accumulation levels	Low	High
Transformation methods	Electroporation, biolistic, glass beads, silicon whiskers	Biolistic
Integration mode	Non-homologous end joining	Homologous recombination

**Table 3:** Summary information of recombinant proteins manufactured in several important microalgae.

Microalgae	Transformation Method	Expressed Genes	Promoters	Selective Marker Genes	Products	Expression Location (Chloroplast/Nucleus)	Product Content or Activity	References
*Amphidinium* sp.	SiC whiskers	*Gus*	CaMV 35S	*nptII* gene (neomycin resistance), *hpt* gene (hygromycin resistance)	β-glucoronidase	Nucleus	–	[88]
*C. reinhardtii*	Particle bombardment	*lsc*	atpA or rbcL	a 16S ribosomal gene (spectinomycin resistance)	Anti-HSV glycoprotein D Isc	Chloroplast	>1% of TSP	[151]
*C. reinhardtii*	Particle bombardment	*gelonin*	psbA	*aphA*6 (kanamycin resistance)	Anti-CD22-gelonin sc	Chloroplast	0.1%–0.2% of TSP	[152]
*C. reinhardtii*	Particle bombardment	*Exotoxin A*	psbA	*aphA*6	Anti-CD22-ETA sc	Chloroplast	0.2%–0.3% of TSP	[153]
*C. reinhardtii*	Particle bombardment	*VP1*, *CTB*	atpA	*aadA* (spectinomycin resistance)	VP1-CTB	Chloroplast	3% of TSP	[154]
*C. reinhardtii*	Glass beads method	*E7GGG*	psbD	*aadA*	E7GGG	Chloroplast	0.12% of TSP	[155]
*C. reinhardtii*	Particle bombardment	*VEGF*	psbA	*aphA*6	VEGF	Chloroplast	2% of TSP	[156]
*C. reinhardtii*	Particle bombardment	*HMGB1*	psbA	*aphA*6	HMGB1	Chloroplast	2.5% of TSP	[156]
*C. reinhardtii*	Particle bombardment	*14FN3*	psbA	*aphA*6	14FN3	Chloroplast	3% of TSP	[156]
*C. reinhardtii*	Particle bombardment	*metallothionein*-*2*	psbA	*aadA*	Metallothionein-2	Chloroplast	–	[157]
*C. reinhardtii*	Particle bombardment	*Strail*	atpA	*aadA*	TRAIL	Chloroplast	0.43%–0.67% of TSP	[158]
*C. reinhardtii*	Particle bombardment	*apcA* and *apcB*	psbA	*aadA*	Allophycocyanin	Chloroplast	2%–3% of TSP	[159]
*C. reinhardtii*	–	IgG1 *lc, hc*	psbA	*aadA*	Anti-PA 83 anthrax IgG1	Chloroplast	100 μg/g of dry algal biomass	[160]
*C. reinhardtii*	Glass beads method	*crEpo*	HSP70A/RBCS2	*ARG*7	Erythropoietin	Nucleus	100 μg /L	[161]
*C. ellipsoidea*	Electroporation	*mNP-1*	Ubi1	*Npt*II and *NR* genes	mNP-1	Nucleus	11.42 mg/L	[120]
*C. reinhardtii*	Particle Bombardment	*m-saa*	psbA	–	M-SAA (bovine mammary-associated serum amyloid)	Chloroplast	5% of TSP	[162]
*C. reinhardtii*	Particle bombardment	*E2*	atpA	*aadA*	Swine fever virus (CSFV) structural protein	Chloroplast	1.5%–2% of TSP	[163]
*C. reinhardtii*	Particle bombardment	*VP28*	psbA	*aadA*	VP28	Chloroplast	21% of TCP (about 42% of TSP)	[164]
*C. reinhardtii*	Particle bombardment	*hGAD65*	rbcL	a 16S ribosomal gene	hGAD65	Chloroplast	0.3% of TSP	[165]
*C. reinhardtii*	Particle bombardment	*CTB*, *D2*	rbcL	aadA	CTB-D2	Chloroplast	0.7% of TSP	[166]
*C. reinhardtii*	Glass beads method	*PfMSP*_1-19_	HSP70A/RbcS2	*AphVIII* gene (paromomycin resistance)	GBSS-PfMSP_1–19_	Nucleus	–	[167]
*C. reinhardtii*	Glass beads method	*PbAMA*_1-C_	HSP70A/RbcS2	*AphVIII* gene	GBSS-PbAMA_1-C_	Nucleus	–	[167]
*C. reinhardtii*	Particle bombardment	*Pfs25*	psbA	*kanR* (a kanamycin resistance cassette)	Pfs25	Chloroplast	–	[168]
*C. reinhardtii*	Particle bombardment	*Pfs28*	psbA	*kanR*	Pfs28	Chloroplast	–	[168]
*C. reinhardtii*	Particle bombardment	*c.r.pfs48/45*	psbA/psbD	–	c.r.Pfs48/45	Chloroplast	–	[169]
*C. reinhardtii*	Particle bombardment	*Cr.ctxB-pfs25*	psbA	*kanR*	Cr.CtxB-Pfs25	Chloroplast	–	[170]
*C. reinhardtii*	Particle bombardment	*appA*	atpA	–	AppA phytase	Chloroplast	–	[171]
*C. reinhardtii*	Electroporation	*xyn1*	PAR4 (hsp70A and the rbcs2)	*aph*7 gene (hygromycin B resistance), *sh-ble* gene (zeocin resistance)	β-1,4-endoxylanase	Nucleus	–	[172]
*C. reinhardtii*	LiAc/PEG	*fGH*	CaMV 35S	*Sh ble* gene (phleomycin resistance)	Flounder growth hormone	Nucleus	420 μg of fGH protein per liter of culture	[128]
*C. reinhardtii*	Glass beads method	*human Sep15*	Hsp70-RBCS2	*ble* gene (zeocin resistance)	Human Sep15 protein	Nucleus	–	[173]
*D. salina*	Electroporation	*HBsAg*	ubiquitin-Ω	*CAT* gene (chloramphenicol resistance)	HBsAg	Nucleus	1.6–3.1 ng/mg	[133]
*D. salina*	Glass beads method	*VP28*	Ubi1-Ω	–	VP28	Nucleus	3 ng/mg	[174]
*D.tertiolecta*	Particle bombardment	*xylanase/α-galactosidase/phytase*	psbD	Erythromycin esterase (erythromycin resistance)	Xylanase/α-galactosidase/phytase	Chloroplast	–	[106]
*L. amoebiformis*	Particle bombardment	*RbcS*	LarbcS1 promoter	–	Rubisco small subunit (RbcS) protein	Nucleus	–	[175]
*Porphyridium* sp.	Particle bombardment	*AHAS* (*W492S*)	Endogenous AHAS promoter	*AHAS*	acetohydroxyacid synthase	Chloroplast	–	[98]
*P.tricornutum*	Particle bombardment	IgG *LC*, *HC*	Nitrate reductase promoter	–	Monoclonal human IgG antibody against HBsAg	Chloroplast	8.7% of TSP, 21 mg/g of dry algal biomass	[123]
*S.microadriaticum*	SiC whiskers	*Gus*	CaMV 35S	*nptII* gene, *hpt* gene	β-glucoronidase	Nucleus	–	[88]
*C. reinhardtii*	Agrobacterium	*HBcAgII*	CaMV 35S	*NptII* (kanamycin resistance)	HBcAg-GS-AgII-GS-HBcAg	Chloroplast	0.02%–0.05% of TSP	[176]
*C. reinhardtii*	Glass beads method	*P24*	*PSAD* promoter	*aphVIII* gene (paromomycin resistance)	subunit of HIV-1 viral particles	Nucleus	0.25% of the total cellular protein	[177]
*C. reinhardtii*	Particle bombardment	*CTB:p210*	*atpA*	*aadA* gene (spectinomycin resistance)	the p210 epitope from ApoB100	Chloroplast	up to 60 μg/g of fresh weight biomass	[178]

*C. reinhardtii* represents *Chlamydomonas reinhardtii*; *C. ellipsoidea* represents *Chlorella ellipsoidea*; *D. salina* represents *Dunaliella salina*; *S. microadriaticum* represents *Symbiodinium microadriaticum*; *L. amoebiformis* represents *Lotharella amoebiformis*; *P. tricornutum* represents *Phaeodactylum tricornutum*; TSP represents total soluble proteins.

**Table 4:** Preclinical trials of recombinant proteins expressed in microalgae.

Recombinant Proteins	Preclinical Trials	Activity Analysis	References
Anti-CD22-ETA sc	Compared with mice treated with the antibody deficient immune toxin, mice treated with immunotoxin produced by algae can significantly inhibit the propagation of tumor, suggesting immunotoxins expressed in algae significantly affect tumor progression in animal experiments.	–	[153]
VP1-CTB	The CTB-VP1, fusion protein expressed in *C. reinhardtii* chloroplast, exhibited significant binding affinity to GM1-ganglioside.	Southern blot, Western blot, ELISA	[154]
E7GGG	E7GGG expressed in *Chlamydomonas* elicited tumor protection in 60% of mice. Mice vaccinated with the purified E7GGG-FLAG protein were 100% tumor-free after 9 weeks.	Western blot, ELISA	[155]
E2	Responsing against CSFV, E2 can induce significant serum antibody by subcutaneous immunization, but oral immunization resulted in no detectable serum response.	Southern blot, Western blot	[163]
hGAD65	Algal-derived hGAD65 had higher binding capacity to diabetic serum samples and can stimulate proliferation of splenic T cells from NOD mice *in vitro*, demonstrating its antigenic and immunogenic characteristics.	Western blot	[165]
CTB-D2	Oral vaccination of CTB-D2 to mice induced responses of specific systemic and mucosal immune against *S. aureus*.	–	[166]
GBSS-pfMSP_1-19_ GBSS-pfAMA_1-C_	The starch antigens accumulated in the chloroplast can protect against lethal *P. berghei* infection in mice.	Western blot	[167]
Cr.CtxB-Pfs25	Oral vaccination of Cr.CtxB-Pfs25-producing algae to mice elicited IgG and IgA antibodies to CTB, and IgG antibodies to Pfs25.	ELISA	[170]
AppA phytase	AppA-producing algae can significantly reduce the phytate content and increase the content of inorganic phosphate content in chick manure.	–	[171]
fGH	Flounder fry fed with transgenic *Chlorella* increased by 25% within 30 days in length and width.	Southern blot, immunoblot	[128]
VP28	Survival rates of crayfish vaccinated by Ds-VP28 were significantly higher (59% mortality) than the positive control groups and Ds empty control (100% mortality).	Western blot, ELISA	[174]
p210 epitope from ApoB100	The immunogenic activity was tested by orally administration in BALB/c mice, and elicited anti-p210 serum antibodies, which can last for at least 80 days.	Western blot, ELISA	[178]

## References

[1] Demain A.L., Vaishnav P. (2009). Production of recombinant proteins by microbes and higher organisms. Biotechnol. Adv..

[2] Ferrer-Miralles N., Domingo-Espín J., Corchero J.L., Vázquez E., Villaverde A. (2009). Microbial factories for recombinant pharmaceuticals. Microb. Cell Fact..

[3] Corchero J.L., Gasser B., Resina D., Smith W., Parrilli E., Vázquez F., Abasolo I., Giuliani M., Jäntti J., Ferrer P. (2013). Unconventional microbial systems for the cost-efficient production of high-quality protein therapeutics. Biotechnol. Adv..

[4] Sanchez S., Demain A.L. (2010). Enzymes and bioconversions of industrial, pharmaceutical, and biotechnological significance. Org. Process Res. Dev..

[5] Cereghino G.P.L., Cregg J.M. (1999). Applications of yeast in biotechnology: Protein production and genetic analysis. Curr. Opin. Biotechnol..

[6] Swartz J.R. (2001). Advances in *Escherichia coli* production of therapeutic proteins. Curr. Opin. Biotechnol..

[7] Fischer R., Liao Y.-C., Hoffmann K., Schillberg S., Emans N. (1999). Molecular farming of recombinant antibodies in plants. Biol. Chem..

[8] Kelley B. (2001). Biochemical engineering: Bioprocessing of therapeutic proteins. Curr. Opin. Biotechnol..

[9] Chu L., Robinson D.K. (2001). Industrial choices for protein production by large-scale cell culture. Curr. Opin. Biotechnol..

[10] Jänne J., Alhonen L., Hyttinen J.-M., Peura T., Tolvanen M., Korhonen V.-P. (1998). Transgenic bioreactors. Biotechnol. Ann. Rev..

[11] Giddings G., Allison G., Brooks D., Carter A. (2000). Transgenic plants as factories for biopharmaceuticals. Nat. Biotechnol..

[12] Echelard Y. (1996). Recombinant protein production in transgenic animals. Curr. Opin. Biotechnol..

[13] Scott M.R., Will R., Ironside J., Nguyen H.-O.B., Tremblay P., DeArmond S.J., Prusiner S.B. (1999). Compelling transgenetic evidence for transmission of bovine spongiform encephalopathy prions to humans. Proc. Natl. Acad. Sci. USA.

[14] Mason H.S., Arntzen C.J. (1995). Transgenic plants as vaccine production systems. Trends Biotechnol..

[15] Cabanes-Macheteau M., Fitchette-Lainé A.-C., Loutelier-Bourhis C., Lange C., Vine N.D., Ma J.K., Lerouge P., Faye L. (1999). *N*-Glycosylation of a mouse IgG expressed in transgenic tobacco plants. Glycobiology.

[16] Herbers K., Sonnewald U. (1999). Production of new/modified proteins in transgenic plants. Curr. Opin. Biotechnol..

[17] Mason H.S., Lam D., Arntzen C.J. (1992). Expression of hepatitis B surface antigen in transgenic plants. Proc. Natl. Acad. Sci. USA.

[18] De Cosa B., Moar W., Lee S.-B., Miller M., Daniell H. (2001). Overexpression of the Bt CRY2Aa2 operon in chloroplasts leads to formation of insecticidal crystals. Nat. Biotechnol..

[19] Sijmons P.C., Dekker B.M., Schrammeijer B., Verwoerd T.C., van den Elzen P.J., Hoekema A. (1990). Production of correctly processed human serum albumin in transgenic plants. Nat. Biotechnol..

[20] Cramer C., Boothe J., Oishi K. (2000). Transgenic plants for therapeutic proteins: Linking upstream and downstream strategies. Plant Biotechnol..

[21] Mor T.S., Sternfeld M., Soreq H., Arntzen C.J., Mason H.S. (2001). Expression of recombinant human acetylcholinesterase in transgenic tomato plants. Biotechnol. Bioeng..

[22] Sheldrake A. (1973). The production of hormones in higher plants. Biol. Rev..

[23] Streatfield S.J., Lane J.R., Brooks C.A., Barker D.K., Poage M.L., Mayor J.M., Lamphear B.J., Drees C.F., Jilka J.M., Hood E.E. (2003). Corn as a production system for human and animal vaccines. Vaccine.

[24] Hiatt A., Caffferkey R., Bowdish K. (1989). Production of antibodies in transgenic plants. Nature.

[25] Ram N., Ayala M., Lorenzo D., Palenzuela D., Herrera L., Doreste V., Pérez M., Gavilondo J.V., Oramas P. (2002). Expression of a single-chain Fv antibody fragment specific for the hepatitis B surface antigen in transgenic tobacco plants. Transgenic Res..

[26] Borisjuk N.V., Borisjuk L.G., Logendra S., Petersen F., Gleba Y., Raskin I. (1999). Production of recombinant proteins in plant root exudates. Nat. Biotechnol..

[27] Fischer R., Emans N. (2000). Molecular farming of pharmaceutical proteins. Transgenic Res..

[28] Arntzen C. (2015). Plant-made pharmaceuticals: From ‘Edible Vaccines’ to Ebola therapeutics. Plant Biotechnol. J..

[29] Jin S., Zhu L., Sherman A., Wang X., Lin S., Kamesh A., Norikane J.H., Streatfield S.J., Herzog R.W., Daniell H. (2015). Low cost industrial production of coagulation factor IX bioencapsulated in lettuce cells for oral tolerance induction in hemophilia B. Biomaterials.

[30] Su J., Sherman A., Doerfler P.A., Byrne B.J., Herzog R.W., Daniell H. (2015). Oral delivery of Acid Alpha Glucosidase epitopes expressed in plant chloroplasts suppresses antibody formation in treatment of Pompe mice. Plant Biotechnol. J..

[31] Chan H.T., Daniell H. (2015). Plant-made oral vaccines against human infectious diseases—Are we there yet?. Plant Biotechnol. J..

[32] Takaiwa F., Wakasa Y., Takagi H., Hiroi T. (2015). Rice seed for delivery of vaccines to gut mucosal immune tissues. Plant Biotechnol. J..

[33] Fox J.L. (2012). First plant-made biologic approved. Nat. Biotechnol..

[34] Daniell H., Streatfield S.J., Rybicki E.P. (2015). Advances in molecular farming: Key technologies, scaled up production and lead targets. Plant Biotechnol. J..

[35] Quist D., Chapela I.H. (2001). Transgenic DNA introgressed into traditional maize landraces in Oaxaca, Mexico. Nature.

[36] Rieger M.A., Lamond M., Preston C., Powles S.B., Roush R.T. (2002). Pollen-mediated movement of herbicide resistance between commercial canola fields. Science.

[37] Griesbeck C., Kobl I., Heitzer M. (2006). *Chlamydomonas reinhardtii*: A protein expression system for pharmaceutical and biotechnological proteins. Mol. Biotechnol..

[38] Potvin G., Zhang Z. (2010). Strategies for high-level recombinant protein expression in transgenic microalgae: A review. Biotechnol. Adv..

[39] Wang S.-K., Hu Y.-R., Wang F., Stiles A.R., Liu C.-Z. (2014). Scale-up cultivation of *Chlorella ellipsoidea* from indoor to outdoor in bubble column bioreactors. Bioresour. Technol..

[40] Clarke A.R. (1995). Photosynthesis fifth edition: By D O Hall and K K Rao, pp 211. Cambridge University Press, Cambridge. 1994. £9.95/$14.95 ISBN 0-521-43622-2. Biochem. Educ..

[41] Chisti Y. (2007). Biodiesel from microalgae. Biotechnol. Adv..

[42] Olaizola M. (2003). Commercial development of microalgal biotechnology: From the test tube to the marketplace. Biomol. Eng..

[43] Morris H.J., Almarales A., Carrillo O., Bermúdez R.C. (2008). Utilisation of *Chlorella vulgaris* cell biomass for the production of enzymatic protein hydrolysates. Bioresour. Technol..

[44] Shimizu Y., Li B., Proksch P., Muller W.E.G. (2006). Microalgae as a source of bioactive molecules: Special problems and methodology. Frontiers in Marine Biotechnology.

[45] Borowitzka M.A. (1999). Commercial production of microalgae: Ponds, tanks, tubes and fermenters. J. Biotechnol..

[46] Kumar R.R., Rao P.H., Subramanian V.V., Sivasubramanian V. (2011). Enzymatic and non-enzymatic antioxidant potentials of *Chlorella vulgaris* grown in effluent of a confectionery industry. J. Food Sci. Technol..

[47] Chisti Y., Posten C., Walter C. (2012). Microalgal Biotechnology: Potential and Production.

[48] Das P., Aziz S.S., Obbard J.P. (2011). Two phase microalgae growth in the open system for enhanced lipid productivity. Renew. Energ..

[49] Richmond A. (2008). Handbook of Microalgal Culture: Biotechnology and Applied Phycology.

[50] Abreu A.P., Fernandes B., Vicente A.A., Teixeira J., Dragone G. (2012). Mixotrophic cultivation of *Chlorella vulgaris* using industrial dairy waste as organic carbon source. Bioresour. Technol..

[51] Basaran P., Rodríguez-Cerezo E. (2008). Plant molecular farming: Opportunities and challenges. J. Serb. Chem. Soc..

[52] Yao J., Weng Y., Dickey A., Wang K.Y. (2015). Plants as factories for human pharmaceuticals: Applications and challenges. Int. J. Mol. Sci..

[53] Chu W.-L. (2012). Biotechnological applications of microalgae. IeJSME.

[54] Kaplan D., Richmond A., Dubinsky Z., Aaronson S. (1986). Algal nutrition. Handbook of Microalgal Mass Culture.

[55] Cunningham A., Maas P. (1978). Time lag and nutrient storage effects in the transient growth response of *Chlamydomonas reinhardii* in nitrogen-limited batch and continuous culture. Microbiology.

[56] Garcia-Ferris C., Rios A., Ascaso C., Moreno J. (1996). Correlated biochemical and ultrastructural changes in nitrogen-starved *Euglena gracilis*. J. Phycol..

[57] Přibyl P., Cepák V., Zachleder V. (2012). Production of lipids in 10 strains of *Chlorella* and *Parachlorella*, and enhanced lipid productivity in *Chlorella vulgaris*. Appl. Microbiol. Biotechnol..

[58] Zachleder V., Brányiková I., Bajpai R., Prokop A., Zappi M. (2014). Starch overproduction by means of algae. Algal Biorefineries.

[59] Spolaore P., Joannis-Cassan C., Duran E., Isambert A. (2006). Commercial applications of microalgae. J. Biosci. Bioeng..

[60] Soletto D., Binaghi L., Lodi A., Carvalho J., Converti A. (2005). Batch and fed-batch cultivations of *Spirulina platensis* using ammonium sulphate and urea as nitrogen sources. Aquaculture.

[61] Guil-Guerrero J., Navarro-Juárez R., López-Martınez J., Campra-Madrid P., Rebolloso-Fuentes M. (2004). Functional properties of the biomass of three microalgal species. J. Food Eng..

[62] Becker W., Richmond A., Richmond A. (2004). Microalgae in human and animal nutrition. Handbook of Microalgal Culture: Biotechnology and Applied Phycology.

[63] Metting F. (1996). Biodiversity and application of microalgae. J. Ind. Microbiol..

[64] Borowitzka M. (1988). Fats, oils and hydrocarbons. Micro-Algal Biotechnology.

[65] Brown M., Jeffrey S., Volkman J., Dunstan G. (1997). Nutritional properties of microalgae for mariculture. Aquaculture.

[66] Tonon T., Harvey D., Larson T.R., Graham I.A. (2002). Long chain polyunsaturated fatty acid production and partitioning to triacylglycerols in four microalgae. Phytochemistry.

[67] Tzovenis I., de Pauw N., Sorgeloos P. (2003). Optimisation of T-ISO biomass production rich in essential fatty acids: I. Effect of different light regimes on growth and biomass production. Aquaculture.

[68] Apt K.E., Behrens P.W. (1999). Commercial developments in microalgal biotechnology. J. Phycol..

[69] Rasoul-Amini S., Ghasemi Y., Morowvat M.H., Mohagheghzadeh A. (2009). PCR amplification of 18S rRNA, single cell protein production and fatty acid evaluation of some naturally isolated microalgae. Food Chem..

[70] Calvin M., Benson A. (1948). The Path of Carbon in Photosynthesis.

[71] Hirose Y., Fujisawa T., Ohtsubo Y., Katayama M., Misawa N., Wakazuki S., Shimura Y., Nakamura Y., Kawachi M., Yoshikawa H. (2016). Complete genome sequence of cyanobacterium *Nostoc* sp. NIES-3756, a potentially useful strain for phytochrome-based bioengineering. J. Biotechnol..

[72] Kaneko T., Tabata S. (1997). Complete genome structure of the unicellular cyanobacterium *Synechocystis* sp. PCC6803. Plant Cell Physiol..

[73] Matsuzaki M., Misumi O., Shin-i T., Maruyama S., Takahara M., Miyagishima S.-Y., Mori T., Nishida K., Yagisawa F., Nishida K. (2004). Genome sequence of the ultrasmall unicellular red alga *Cyanidioschyzon merolae* 10D. Nature.

[74] Armbrust E.V., Berges J.A., Bowler C., Green B.R., Martinez D., Putnam N.H., Zhou S., Allen A.E., Apt K.E., Bechner M. (2004). The genome of the diatom *Thalassiosira pseudonana*: Ecology, evolution, and metabolism. Science.

[75] Merchant S.S., Prochnik S.E., Vallon O., Harris E.H., Karpowicz S.J., Witman G.B., Terry A., Salamov A., Fritz-Laylin L.K., Maréchal-Drouard L. (2007). The *Chlamydomonas* genome reveals the evolution of key animal and plant functions. Science.

[76] Fan J., Ning K., Zeng X., Luo Y., Wang D., Hu J., Li J., Xu H., Huang J., Wan M. (2015). Genomic foundation of starch-to-lipid switch in oleaginous *Chlorella* spp.. Plant Physiol..

[77] Radakovits R., Jinkerson R.E., Fuerstenberg S.I., Tae H., Settlage R.E., Boore J.L., Posewitz M.C. (2012). Draft genome sequence and genetic transformation of the oleaginous alga nannochloropsis gaditana. Nat. Commun..

[78] Fu Y., Luo G.Z., Chen K., Deng X., Yu M., Han D., Hao Z., Liu J., Lu X., Doré L. (2015). *N*^6^-Methyldeoxyadenosine marks active transcription start sites in *Chlamydomonas*. Cell.

[79] Li X., Zhang R., Patena W., Gang S.S., Blum S.R., Ivanova N., Yue R., Robertson J.M., Lefebvre P., Fitz-Gibbon S.T. (2016). An indexed, mapped mutant library enables reverse genetics studies of biological processes in *Chlamydomonas reinhardtii*. Plant Cell.

[80] Yang W., Wittkopp T.M., Li X., Warakanont J., Dubini A., Catalanotti C., Kim R.G., Nowack E.C.M., Mackinder L.C.M., Aksoy M. (2015). Critical role of *Chlamydomonas reinhardtii* ferredoxin-5 in maintaining membrane structure and dark metabolism. Proc. Natl. Acad. Sci. USA.

[81] Dejtisakdi W., Miller S.M. (2016). Overexpression of calvin cycle enzyme fructose 1,6-bisphosphatase in *Chlamydomonas reinhardtii* has a detrimental effect on growth. Algal Res..

[82] Sirikhachornkit A., Vuttipongchaikij S., Suttangkakul A., Yokthongwattana K., Juntawong P., Pokethitiyook P., Kangvansaichol K., Meetam M. (2016). Increasing the triacylglycerol content in *Dunaliella tertiolecta* through isolation of starch-deficient mutants. J. Microbiol. Biotechnol..

[83] Kira N., Ohnishi K., Miyagawa-Yamaguchi A., Kadono T., Adachi M. (2016). Nuclear transformation of the diatom *Phaeodactylum tricornutum* using PCR-amplified DNA fragments by microparticle bombardment. Mar. Genom..

[84] Ferreira-Camargo L.S., Tran M., Beld J., Burkart M.D., Mayfield S.P. (2015). Selenocystamine improves protein accumulation in chloroplasts of eukaryotic green algae. AMB Express.

[85] Gong Y., Hu H., Gao Y., Xu X., Gao H. (2011). Microalgae as platforms for production of recombinant proteins and valuable compounds: Progress and prospects. J. Ind. Microbiol. Biotechnol..

[86] Kindle K., Rochaix J.-D., Goldschmidt-Clermont M., Merchant S. (2004). Nuclear transformation: Technology and applications. The Molecular Biology of Chloroplasts and Mitochondria in Chlamydomonas.

[87] Dunahay T. (1993). Transformation of *Chlamydomonas reinhardtii* with silicon carbide whiskers. Biotechniques.

[88] Te M.R., Miller D.J. (1998). Genetic transformation of dinoflagellates (*Amphidinium* and *Symbiodinium*): Expression of GUS in microalgae using heterologous promoter constructs. Plant J..

[89] Shimogawara K., Fujiwara S., Grossman A., Usuda H. (1998). High-efficiency transformation of *Chlamydomonas reinhardtii* by electroporation. Genetics.

[90] Hayashi M., Hirono M., Kamiya R. (2001). Recovery of flagellar dynein function in a *Chlamydomonas* actin/dynein-deficient mutant upon introduction of muscle actin by electroporation. Cell Motil. Cytoskelet..

[91] Henry E.C., Meints R.H. (1994). Recombinant viruses as transformation vectors of marine macroalgae. J. Appl. Phycol..

[92] Van Etten J.L., Meints R.H. (1999). Giant viruses infecting algae. Annu. Rev. Microbiol..

[93] Kojima H., Kawata Y., Kojima H., Lee Y.K. (2001). A mini-transposon/transposase complex as a new tool for the genetic transformation of microalgae. Photosynthetic Microorganisms in Environmental Biotechnology.

[94] Apt K.E., Grossman A., Kroth-Pancic P. (1996). Stable nuclear transformation of the diatom *Phaeodactylum tricornutum*. Mol. Gen. Genet..

[95] Dunahay T.G., Jarvis E.E., Roessler P.G. (1995). Genetic transformation of the diatoms *Cyclotella cryptica* and *Navicula saprophila*. J. Phycol..

[96] Falciatore A., Casotti R., Leblanc C., Abrescia C., Bowler C. (1999). Transformation of nonselectable reporter genes in marine diatoms. Mar. Biotechnol..

[97] Zaslavskaia L.A., Lippmeier J.C., Kroth P.G., Grossman A.R., Apt K.E. (2000). Transformation of the diatom *Phaeodactylum tricornutum* (bacillariophyceae) with a variety of selectable marker and reporter genes. J. Phycol..

[98] Lapidot M., Raveh D., Sivan A., Arad S.M., Shapira M. (2002). Stable chloroplast transformation of the unicellular red alga *Porphyridium* species. Plant Physiol..

[99] Boynton J.E., Gillham N.W., Harris E.H., Hosler J.P., Johnson A.M., Jones A.R., Randolph-Anderson B.L., Robertson D., Klein T.M., Shark K.B. (1988). Chloroplast transformation in *Chlamydomonas* with high velocity microprojectiles. Science.

[100] Doetsch N.A., Favreau M.R., Kuscuoglu N., Thompson M.D., Hallick R.B. (2001). Chloroplast transformation in *Euglena gracilis*: Splicing of a group III twintron transcribed from a transgenic *psbK* operon. Curr. Genet..

[101] Randolph-Anderson B.L., Boynton J.E., Gillham N.W., Harris E.H., Johnson A.M., Dorthu M.-P., Matagne R.F. (1993). Further characterization of the respiratory deficient *dum-1* mutation of *Chlamydomonas reinhardtii* and its use as a recipient for mitochondrial transformation. Mol. Gen. Genet..

[102] Fernández E., Schnell R., Ranum L., Hussey S.C., Silflow C.D., Lefebvre P.A. (1989). Isolation and characterization of the nitrate reductase structural gene of *Chlamydomonas reinhardtii*. Proc. Natl. Acad. Sci. USA.

[103] Debuchy R., Purton S., Rochaix J. (1989). The argininosuccinate lyase gene of *Chlamydomonas reinhardtii*: An important tool for nuclear transformation and for correlating the genetic and molecular maps of the ARG7 locus. EMBO J..

[104] Shrager J., Hauser C., Chang C.-W., Harris E.H., Davies J., McDermott J., Tamse R., Zhang Z., Grossman A.R. (2003). *Chlamydomonas reinhardtii* genome project. A guide to the generation and use of the cDNA information. Plant Physiol..

[105] Dawson H.N., Burlingame R., Cannons A.C. (1997). Stable transformation of *Chlorella*: Rescue of nitrate reductase-deficient mutants with the nitrate reductase gene. Curr. Microbiol..

[106] Georgianna D.R., Hannon M.J., Marcuschi M., Wu S., Botsch K., Lewis A.J., Hyun J., Mendez M., Mayfield S.P. (2013). Production of recombinant enzymes in the marine alga *Dunaliella tertiolecta*. Algal Res..

[107] Gutierrez C.L., Gimpel J., Escobar C., Marshall S.H., Henríquez V. (2012). Chloroplast genetic tool for the green microalgae *Haematococcus pluvialis* (chlorophyceae, volvocales). J. Phycol..

[108] Fischer H., Robl I., Sumper M., Kröger N. (1999). Targeting and covalent modification of cell wall and membrane proteins heterologously expressed in the diatom *Cylindrotheca fusiformis* (bacillariophyceae). J. Phycol..

[109] Poulsen N., Chesley P.M., Kröger N. (2006). Molecular genetic manipulation of the diatom *Thalassiosira pseudonana* (bacillariophyceae). J. Phycol..

[110] Miyagawa-Yamaguchi A., Okami T., Kira N., Yamaguchi H., Ohnishi K., Adachi M. (2011). Stable nuclear transformation of the diatom *Chaetoceros* sp.. Phycol. Res..

[111] Walker T.L., Purton S., Becker D.K., Collet C. (2005). Microalgae as bioreactors. Plant Cell Rep..

[112] Kindle K.L., Schnell R.A., Fernández E., Lefebvre P.A. (1989). Stable nuclear transformation of *Chlamydomonas* using the *Chlamydomonas* gene for nitrate reductase. J. Cell Biol..

[113] Schiedlmeier B., Schmitt R., Müller W., Kirk M.M., Gruber H., Mages W., Kirk D.L. (1994). Nuclear transformation of *Volvox carteri*. Proc. Natl. Acad. Sci. USA.

[114] Sun Y., Gao X., Li Q., Zhang Q., Xu Z. (2006). Functional complementation of a nitrate reductase defective mutant of a green alga *Dunaliella viridis* by introducing the nitrate reductase gene. Gene.

[115] Cerutti H., Johnson A.M., Gillham N.W., Boynton J.E. (1997). A eubacterial gene conferring spectinomycin resistance on *Chlamydomonas reinhardtii*: Integration into the nuclear genome and gene expression. Genetics.

[116] Sizova I., Fuhrmann M., Hegemann P. (2001). A *Streptomyces rimosus aphVIII* gene coding for a new type phosphotransferase provides stable antibiotic resistance to *Chlamydomonas reinhardtii*. Gene.

[117] Stevens D.R., Purton S., Rochaix J.-D. (1996). The bacterial phleomycin resistance gene *ble* as a dominant selectable marker in *Chlamydomonas*. Mol. Gen. Genet..

[118] Kovar J.L., Zhang J., Funke R.P., Weeks D.P. (2002). Molecular analysis of the acetolactate synthase gene of *Chlamydomonas reinhardtii* and development of a genetically engineered gene as a dominant selectable marker for genetic transformation. Plant J..

[119] Hallmann A., Rappel A. (1999). Genetic engineering of the multicellular green alga *Volvox*: A modified and multiplied bacterial antibiotic resistance gene as a dominant selectable marker. Plant J..

[120] Bai L.-L., Yin W.-B., Chen Y.-H., Niu L.-L., Sun Y.-R., Zhao S.-M., Yang F., Wang R.R.-C., Wu Q., Zhang X. (2013). A new strategy to produce a defensin: Stable production of mutated NP-1 in nitrate reductase-deficient *Chlorella ellipsoidea*. PLoS ONE.

[121] Zhang J., Hao Q., Bai L., Xu J., Yin W., Song L., Xu L., Guo X., Fan C., Chen Y. (2014). Overexpression of the soybean transcription factor GmDof4 significantly enhances the lipid content of *Chlorella ellipsoidea*. Biotechnol. Biofuels.

[122] Miyagawa A., Okami T., Kira N., Yamaguchi H., Ohnishi K., Adachi M. (2009). High efficiency transformation of the diatom *Phaeodactylum tricornutum* with a promoter from the diatom *Cylindrotheca fusiformis*. Phycol. Res..

[123] Hempel F., Lau J., Klingl A., Maier U.G. (2011). Algae as protein factories: Expression of a human antibody and the respective antigen in the diatom *Phaeodactylum tricornutum*. PLoS ONE.

[124] Poulsen N., Kröger N. (2005). A new molecular tool for transgenic diatoms. FEBS J..

[125] Gonzalez N.H., Felsner G., Schramm F.D., Klingl A., Maier U.-G., Bolte K. (2011). A single peroxisomal targeting signal mediates matrix protein import in diatoms. PLoS ONE.

[126] Hempel F., Maier U.G. (2012). An engineered diatom acting like a plasma cell secreting human lgG antibodies with high efficiency. Microb. Cell Fact..

[127] Stork S., Moog D., Przyborski J.M., Wilhelmi I., Zauner S., Maier U.G. (2012). Distribution of the selma translocon in secondary plastids of red algal origin and predicted uncoupling of ubiquitin-dependent translocation from degradation. Eukaryot. Cell.

[128] Kim D.-H., Kim Y.T., Cho J.J., Bae J.-H., Hur S.-B., Hwang I., Choi T.-J. (2002). Stable integration and functional expression of flounder growth hormone gene in transformed microalga, *Chlorella ellipsoidea*. Mar. Biotechnol..

[129] Jarvis E.E., Brown L.M. (1991). Transient expression of firefly luciferase in protoplasts of the green alga *Chlorella ellipsoidea*. Curr. Genet..

[130] Chen Y., Wang Y., Sun Y., Zhang L., Li W. (2001). Highly efficient expression of rabbit neutrophil peptide-1 gene in *Chlorella ellipsoidea* cells. Curr. Genet..

[131] Hawkins R.L., Nakamura M. (1999). Expression of human growth hormone by the eukaryotic alga, *Chlorella*. Curr. Microbiol..

[132] Kathiresan S., Chandrashekar A., Ravishankar G., Sarada R. (2009). *Agrobacterium*-mediated transformation in the green alga *Haematococcus pluvialis* (Chlorophyceae, Volvocales). J. Phycol..

[133] Geng D., Wang Y., Wang P., Li W., Sun Y. (2003). Stable expression of hepatitis B surface antigen gene in *Dunaliella salina* (chlorophyta). J. Appl. Phycol..

[134] Feng S., Xue L., Liu H., Lu P. (2009). Improvement of efficiency of genetic transformation for *Dunaliella salina* by glass beads method. Mol. Biol. Rep..

[135] Chai X.-J., Chen H.-X., Xu W.-Q., Xu Y.-W. (2013). Expression of soybean kunitz trypsin inhibitor gene *SKTI* in *Dunaliella salina*. J. Appl. Phycol..

[136] Seo S., Jeon H., Hwang S., Jin E.S., Chang K.S. (2015). Development of a new constitutive expression system for the transformation of the diatom *Phaeodactylum tricornutum*. Algal Res..

[137] Maruyama M., Horáková I., Honda H., Xing X.H., Shiragami N., Unno H. (1994). Introduction of foreign DNA into *Chlorella saccharophila* by electroporation. Biotechnol. Tech..

[138] Yang A., Bai L., Chen Y., Hu Z. (2009). The expression of NP-1 in *Chlorella ellipsoidea*.

[139] Wang Y., Chen Y., Zhang X., Wang P., Geng D., Zhao S., Zhang L., Sun Y. (2005). Isolation and characterization of a nitrate reductase deficient mutant of *Chlorella ellipsoidea* (Chlorophyta). J. Appl. Phycol..

[140] Niu Y.F., Zhang M.H., Xie W.H., Li J.N., Gao Y.F., Yang W.D., Liu J.S., Li H.Y. (2011). A new inducible expression system in a transformed green alga, *Chlorella vulgaris*. Genet. Mol. Res..

[141] Cerutti H., Johnson A.M., Gillham N.W., Boynton J.E. (1997). Epigenetic silencing of a foreign gene in nuclear transformants of *Chlamydomonas*. Plant Cell.

[142] Cerutti H., Ma X., Msanne J., Repas T. (2011). RNA-mediated silencing in algae: Biological roles and tools for analysis of gene function. Eukaryot. Cell.

[143] Kim E.J., Ma X., Cerutti H. (2015). Gene silencing in microalgae: Mechanisms and biological roles. Bioresour. Technol..

[144] Doron L., Segal N.A., Shapira M., Wilhelm R.A., Geisler K. (2016). Transgene expression in microalgae—From tools to applications. Front. Plant Sci..

[145] El-Sheekh M. (1999). Stable transformation of the intact cells of *Chlorella kessleri* with high velocity microprojectiles. Biol. Plant..

[146] Ahmed E.G., Sung Kwon L., Sang M.S., Seung H.Y., Gyuhwa C. (2015). Development of stable marker-free nuclear transformation strategy in the green microalga *Chlorella vulgaris*. Afr. J. Biotechnol..

[147] Pulz O., Scheibenbogen K., Groß W., Rehm H.-J., Reed G. (2008). Biotechnology with cyanobacteria and microalgae. Biotechnology Set.

[148] Dunahay T.G., Jarvis E.E., Dais S.S., Roessler P.G. (1996). Manipulation of microalgal lipid production using genetic engineering. Appl. Biochem. Biotechnol..

[149] Cai X.-H., Brown C., Adhiya J., Traina S.J., Sayre R.T. (1999). Growth and heavy metal binding properties of transgenic *Chlamydomonas* expressing a foreign metallothionein gene. Int. J. Phytoremediat..

[150] Siripornadulsil S., Traina S., Verma D.P.S., Sayre R.T. (2002). Molecular mechanisms of proline-mediated tolerance to toxic heavy metals in transgenic microalgae. Plant Cell.

[151] Mayfield S.P., Franklin S.E., Lerner R.A. (2003). Expression and assembly of a fully active antibody in algae. Proc. Natl. Acad. Sci. USA.

[152] Tran M., Henry R.E., Siefker D., Van C., Newkirk G., Kim J., Bui J., Mayfield S.P. (2013). Production of anti-cancer immunotoxins in algae: Ribosome inactivating proteins as fusion partners. Biotechnol. Bioeng..

[153] Tran M., Van C., Barrera D.J., Pettersson P.L., Peinado C.D., Bui J., Mayfield S.P. (2013). Production of unique immunotoxin cancer therapeutics in algal chloroplasts. Proc. Natl. Acad. Sci. USA.

[154] Sun M., Qian K., Su N., Chang H., Liu J., Shen G. (2003). Foot-and-mouth disease virus VP1 protein fused with cholera toxin B subunit expressed in *Chlamydomonas reinhardtii* chloroplast. Biotechnol. Lett..

[155] Demurtas O.C., Massa S., Ferrante P., Venuti A., Franconi R., Giuliano G. (2013). A *Chlamydomonas*-derived human papillomavirus 16 E7 vaccine induces specific tumor protection. PLoS ONE.

[156] Rasala B.A., Muto M., Lee P.A., Jager M., Cardoso R.M., Behnke C.A., Kirk P., Hokanson C.A., Crea R., Mendez M. (2010). Production of therapeutic proteins in algae, analysis of expression of seven human proteins in the chloroplast of *Chlamydomonas reinhardtii*. Plant Biotechnol. J..

[157] Zhang Y.K., Shen G.F., Ru B.G. (2006). Survival of human metallothionein-2 transplastomic *Chlamydomonas reinhardtii* to ultraviolet B exposure. Acta Biochim. Biophys. Sin..

[158] Yang Z., Chen F., Li D., Zhang Z., Liu Y., Zheng D., Wang Y., Shen G. (2006). Expression of human soluble TRAIL in *Chlamydomonas reinhardtii* chloroplast. Chin. Sci. Bull..

[159] Su Z.L., Qian K.X., Tan C.P., Meng C.X., Qin S. (2005). Recombination and heterologous expression of allophycocyanin gene in the chloroplast of *Chlamydomonas reinhardtii*. Acta Biochim. Biophys. Sin..

[160] Tran M., Zhou B., Pettersson P.L., Gonzalez M.J., Mayfield S.P. (2009). Synthesis and assembly of a full-length human monoclonal antibody in algal chloroplasts. Biotechnol. Bioeng..

[161] Eichler-Stahlberg A., Weisheit W., Ruecker O., Heitzer M. (2009). Strategies to facilitate transgene expression in *Chlamydomonas reinhardtii*. Planta.

[162] Manuell A.L., Beligni M.V., Elder J.H., Siefker D.T., Tran M., Weber A., McDonald T.L., Mayfield S.P. (2007). Robust expression of a bioactive mammalian protein in *Chlamydomonas* chloroplast. Plant Biotechnol. J..

[163] He D.-M., Qian K.-X., Shen G.-F., Zhang Z.-F., Yi-Nü L., Su Z.-L., Shao H.-B. (2007). Recombination and expression of classical swine fever virus (CSFV) structural protein E2 gene in *Chlamydomonas reinhardtii* chroloplasts. Colloids Surf. B Biointerfaces.

[164] Surzycki R., Greenham K., Kitayama K., Dibal F., Wagner R., Rochaix J.-D., Ajam T., Surzycki S. (2009). Factors effecting expression of vaccines in microalgae. Biologicals.

[165] Wang X., Brandsma M., Tremblay R., Maxwell D., Jevnikar A.M., Huner N., Ma S. (2008). A novel expression platform for the production of diabetes-associated autoantigen human glutamic acid decarboxylase (hGAD65). BMC Biotechnol..

[166] Dreesen I.A., Charpin-El Hamri G., Fussenegger M. (2010). Heat-stable oral alga-based vaccine protects mice from *Staphylococcus aureus* infection. J. Biotechnol..

[167] Dauvillée D., Delhaye S., Gruyer S., Slomianny C., Moretz S.E., d’Hulst C., Long C.A., Ball S.G., Tomavo S. (2010). Engineering the chloroplast targeted malarial vaccine antigens in Chlamydomonas starch granules. PLoS ONE.

[168] Gregory J.A., Li F., Tomosada L.M., Cox C.J., Topol A.B., Vinetz J.M., Mayfield S. (2012). Algae-produced Pfs25 elicits antibodies that inhibit malaria transmission. PLoS ONE.

[169] Jones C.S., Luong T., Hannon M., Tran M., Gregory J.A., Shen Z., Briggs S.P., Mayfield S.P. (2013). Heterologous expression of the C-terminal antigenic domain of the malaria vaccine candidate Pfs48/45 in the green algae *Chlamydomonas reinhardtii*. Appl. Microbiol. Biotechnol..

[170] Gregory J.A., Topol A.B., Doerner D.Z., Mayfield S. (2013). Alga-produced cholera toxin-Pfs25 fusion proteins as oral vaccines. Appl. Environ. Microbiol..

[171] Yoon S.-M., Kim S.Y., Li K.F., Yoon B.H., Choe S., Kuo M.M.-C. (2011). Transgenic microalgae expressing *Escherichia coli* AppA phytase as feed additive to reduce phytate excretion in the manure of young broiler chicks. Appl. Microbiol. Biotechnol..

[172] Rasala B.A., Lee P.A., Shen Z., Briggs S.P., Mendez M., Mayfield S.P. (2012). Robust expression and secretion of xylanase1 in *Chlamydomonas reinhardtii* by fusion to a selection gene and processing with the FMDV 2A peptide. PLoS ONE.

[173] Hou Q., Qiu S., Liu Q., Tian J., Hu Z., Ni J. (2013). Selenoprotein-transgenic *Chlamydomonas reinhardtii*. Nutrients.

[174] Feng S., Feng W., Zhao L., Gu H., Li Q., Shi K., Guo S., Zhang N. (2014). Preparation of transgenic *Dunaliella salina* for immunization against white spot syndrome virus in crayfish. Arch. Virol..

[175] Hirakawa Y., Ishida K.I. (2010). Internal plastid-targeting signal found in a RubisCO small subunit protein of a chlorarachniophyte alga. Plant J..

[176] Soria-Guerra R.E., Ramírez-Alonso J.I., Ibáñez-Salazar A., Govea-Alonso D.O., Paz-Maldonado L.M.T., Bañuelos-Hernández B., Korban S.S., Rosales-Mendoza S. (2014). Expression of an HBcAg-based antigen carrying angiotensin II in *Chlamydomonas reinhardtii* as a candidate hypertension vaccine. Plant Cell Tissue Organ Cult..

[177] Barahimipour R., Neupert J., Bock R. (2016). Efficient expression of nuclear transgenes in the green alga Chlamydomonas: Synthesis of an HIV antigen and development of a new selectable marker. Plant Mol. Biol..

[178] Beltrán-López J.I., Romero-Maldonado A., Monreal-Escalante E., Bañuelos-Hernández B., Paz-Maldonado L.M., Rosales-Mendoza S. (2016). Chlamydomonas reinhardtii chloroplasts express an orally immunogenic protein targeting the p210 epitope implicated in atherosclerosis immunotherapies. Plant Cell Rep..

[179] Rasala B.A., Mayfield S.P. (2014). Photosynthetic biomanufacturing in green algae; production of recombinant proteins for industrial, nutritional, and medical uses. Photosynth. Res..

[180] Heitzer M., Eckert A., Fuhrmann M., Griesbeck C. (2007). Influence of codon bias on the expression of foreign genes in microalgae. Adv. Exp. Med. Biol..

[181] Specht E.A., Mayfield S.P. (2014). Algae-based oral recombinant vaccines. Front. Microbiol..

[182] Van Hulten M.C., Witteveldt J., Peters S., Kloosterboer N., Tarchini R., Fiers M., Sandbrink H., Lankhorst R.K., Vlak J.M. (2001). The white spot syndrome virus DNA genome sequence. Virology.

[183] Tisch R., Yang X.-D., Singer S.M., Liblau R.S., Fugger L., McDevitt H.O. (1993). Immune response to glutamic acid decarboxylase correlates with insulitis in non-obese diabetic mice. Nature.

[184] Gregory J.A., Mayfield S.P. (2014). Developing inexpensive malaria vaccines from plants and algae. Appl. Microbiol. Biotechnol..

[185] Liu J., Sun Z., Gerken H., Huang J., Jiang Y., Chen F. (2014). Genetic engineering of the green alga *Chlorella zofingiensis*: A modified norflurazon-resistant phytoene desaturase gene as a dominant selectable marker. Appl. Microbiol. Biotechnol..

[186] Miyahara M., Aoi M., Inoue-Kashino N., Kashino Y., Ifuku K. (2013). Highly efficient transformation of the diatom *Phaeodactylum tricornutum* by multi-pulse electroporation. Biosci. Biotechnol. Biochem..

[187] Liu J., Chen F. (2016). Biology and industrial applications of *Chlorella*: Advances and prospects. Adv. Biochem. Eng. Biotechnol..

[188] Shaaltiel Y., Bartfeld D., Hashmueli S., Baum G., Brill-Almon E., Galili G., Dym O., Boldin-Adamsky S.A., Silman I., Sussman J.L. (2007). Production of glucocerebrosidase with terminal mannose glycans for enzyme replacement therapy of gaucher’s disease using a plant cell system. Plant Biotechnol. J..

[189] Hollak C. (2012). An evidence-based review of the potential benefits of taliglucerase alfa in the treatment of patients with Gaucher disease. Core Evid..

[190] Shaaltiel Y., Gingis-Velitski S., Tzaban S., Fiks N., Tekoah Y., Aviezer D. (2015). Plant-based oral delivery of β-glucocerebrosidase as an enzyme replacement therapy for Gaucher’s disease. Plant Biotechnol. J..

[191] Rosalesmendoza S., Angulo C., Meza B. (2015). Food-grade organisms as vaccine biofactories and oral delivery vehicles. Trends Biotechnol..

[192] Santos R.B., Abranches R., Fischer R., Sack M., Holland T. (2016). Putting the spotlight back on plant suspension cultures. Front. Plant Sci..

[193] Tacket C.O., Mason H.S., Losonsky G., Estes M.K., Levine M.M., Arntzen C.J. (2000). Human Immune Responses to a Novel Norwalk Virus Vaccine Delivered in Transgenic Potatoes. J. Infect. Dis..

[194] Yusibov V., Hooper D., Spitsin S., Fleysh N., Kean R., Mikheeva T., Deka D., Karasev A., Cox S., Randall J. (2002). Expression in plants and immunogenicity of plant virus-based experimental rabies vaccine. Vaccine.

[195] Thanavala Y., Mahoney M., Pal S., Scott A., Richter L., Natarajan N., Goodwin P., Arntzen C.J., Mason H.S. (2005). Immunogenicity in humans of an edible vaccine for hepatitis B. Proc. Natl. Acad. Sci. USA.

[196] Hefferon K. (2010). Clinical trials fuel the promise of plant-derived vaccines. Am. J. Clin. Med..

[197] Heinzelmann E. (2015). The management centre innsbruck keeping one step ahead with algae innovation. Chim. Int. J. Chem..

